# Supplementary material for: From sequence to enzyme mechanism using multi-label machine learning
Source: BMC Bioinformatics. 2014 May 19;15:150. doi: 10.1186/1471-2105-15-150 (PMC4229970; doi:10.1186/1471-2105-15-150)
Supplement: Additional file 2 — Java code of ml2db. Additional file ml2db_code.tar.gz contains the Java source code to run the multi-label machine learning experiments and save the results to database. The code’s Javadoc is included. [file 1471-2105-15-150-S2.zip › additional file 2/ml2db/ml2db/doc/main/ml2db/weka/JaccardDistanceStored.html]

JaccardDistanceStored


JavaScript is disabled on your browser.


- Overview
- Package
- Class
- Use
- Tree
- Deprecated
- Index
- Help

- Prev Class
- Next Class

- Frames
- No Frames

- All Classes

- Summary:
- Nested |
- Field |
- Constr |
- Method

- Detail:
- Field |
- Constr |
- Method


main.ml2db.weka

## Class JaccardDistanceStored

- java.lang.Object
- - main.ml2db.weka.JaccardDistanceStored

- All Implemented Interfaces:
  :   java.io.Serializable, weka.core.DistanceFunction, weka.core.OptionHandler

  ---

    

  ```
  public class JaccardDistanceStored
  extends java.lang.Object
  implements weka.core.DistanceFunction, java.io.Serializable
  ```

  This is an implementation of the Jaccard distance which pre-calculates and
  stores the numbers needed. The Jaccard distance = 1 minus the Jaccard
  coefficient. The Jaccard coefficient measures similarity between sample sets,
  and is defined as the size of the intersection divided by the size of the
  union of the sample sets.

  Version:
  :   14 Apr 2013

  Author:
  :   ldf

  See Also:
  :   Serialized Form

- - ### Constructor Summary

    Constructors

    Constructor and Description

    `JaccardDistanceStored(IdMultiLabelInstances idInstances)`
  - ### Method Summary

    Methods

    | Modifier and Type | Method and Description |
    | --- | --- |
    | `double` | `distance(weka.core.Instance a, weka.core.Instance b)` |
    | `double` | `distance(weka.core.Instance first, weka.core.Instance second, double m_cutOffValue)` |
    | `double` | `distance(weka.core.Instance first, weka.core.Instance second, double cutOffValue, weka.core.neighboursearch.PerformanceStats stats)` |
    | `double` | `distance(weka.core.Instance first, weka.core.Instance second, weka.core.neighboursearch.PerformanceStats m_stats)` |
    | `java.lang.String` | `getAttributeIndices()` |
    | `java.util.HashSet<weka.core.Attribute>` | `getFeatureAttributes()` |
    | `IdMultiLabelInstances` | `getIdInstances()` |
    | `java.util.TreeMap<java.lang.String,java.util.TreeSet<java.lang.Integer>>` | `getInstance2NonZeroFeatAttIndexes()` |
    | `weka.core.Instances` | `getInstances()` |
    | `int` | `getIntersection(java.util.TreeSet<java.lang.Integer> a, java.util.TreeSet<java.lang.Integer> b)` Calculate the size of the intersection of set A (feature attributes of instance A) and set B (feature attributes of instance B), that is, the non-zero attributes they have in common. |
    | `boolean` | `getInvertSelection()` |
    | `DistancesStorage` | `getJaccardDistanceStore()` |
    | `java.lang.String[]` | `getOptions()` |
    | `int` | `getUnion(java.util.TreeSet<java.lang.Integer> a, java.util.TreeSet<java.lang.Integer> b)` Calculate the size of the union of set A (feature attributes of instance A) and set B (feature attributes of instance B), that is, all the non-zero attributes the two instances have. |
    | `java.util.Enumeration` | `listOptions()` |
    | `static void` | `main(java.lang.String[] args)` |
    | `void` | `postProcessDistances(double[] distances)` |
    | `java.lang.String` | `saveToFileAllDistances()` Generate and print distances for each instance vs each other instance. |
    | `void` | `setAttributeIndices(java.lang.String value)` |
    | `void` | `setInstances(weka.core.Instances instances)` |
    | `void` | `setInvertSelection(boolean value)` |
    | `void` | `setOptions(java.lang.String[] options)` |
    | `void` | `update(weka.core.Instance instance)` |

    - ### Methods inherited from class java.lang.Object

      `equals, getClass, hashCode, notify, notifyAll, toString, wait, wait, wait`

- - ### Constructor Detail


    - #### JaccardDistanceStored

      ```
      public JaccardDistanceStored(IdMultiLabelInstances idInstances)
      ```
  - ### Method Detail


    - #### distance

      ```
      public double distance(weka.core.Instance a,
                    weka.core.Instance b)
      ```

      **Specified by:**
      :   `distance` in interface `weka.core.DistanceFunction`


    - #### distance

      ```
      public double distance(weka.core.Instance first,
                    weka.core.Instance second,
                    double m_cutOffValue)
      ```

      **Specified by:**
      :   `distance` in interface `weka.core.DistanceFunction`


    - #### distance

      ```
      public double distance(weka.core.Instance first,
                    weka.core.Instance second,
                    double cutOffValue,
                    weka.core.neighboursearch.PerformanceStats stats)
      ```

      **Specified by:**
      :   `distance` in interface `weka.core.DistanceFunction`


    - #### distance

      ```
      public double distance(weka.core.Instance first,
                    weka.core.Instance second,
                    weka.core.neighboursearch.PerformanceStats m_stats)
                      throws java.lang.Exception
      ```

      **Specified by:**
      :   `distance` in interface `weka.core.DistanceFunction`

      Throws:
      :   `java.lang.Exception`


    - #### getAttributeIndices

      ```
      public java.lang.String getAttributeIndices()
      ```

      **Specified by:**
      :   `getAttributeIndices` in interface `weka.core.DistanceFunction`


    - #### getFeatureAttributes

      ```
      public java.util.HashSet<weka.core.Attribute> getFeatureAttributes()
      ```


    - #### getIdInstances

      ```
      public IdMultiLabelInstances getIdInstances()
      ```


    - #### getInstance2NonZeroFeatAttIndexes

      ```
      public java.util.TreeMap<java.lang.String,java.util.TreeSet<java.lang.Integer>> getInstance2NonZeroFeatAttIndexes()
      ```


    - #### getInstances

      ```
      public weka.core.Instances getInstances()
      ```

      **Specified by:**
      :   `getInstances` in interface `weka.core.DistanceFunction`


    - #### getIntersection

      ```
      public int getIntersection(java.util.TreeSet<java.lang.Integer> a,
                        java.util.TreeSet<java.lang.Integer> b)
      ```

      Calculate the size of the intersection of set A (feature attributes of
      instance A) and set B (feature attributes of instance B), that is, the
      non-zero attributes they have in common.

      Parameters:
      :   `a` -
      :   `b` -

      Returns:
      :   the size of the intersection of set A (feature attributes of
          instance A) and set B (feature attributes of instance B), that
          is, the non-zero attributes they have in common.


    - #### getInvertSelection

      ```
      public boolean getInvertSelection()
      ```

      **Specified by:**
      :   `getInvertSelection` in interface `weka.core.DistanceFunction`


    - #### getJaccardDistanceStore

      ```
      public DistancesStorage getJaccardDistanceStore()
      ```


    - #### getOptions

      ```
      public java.lang.String[] getOptions()
      ```

      **Specified by:**
      :   `getOptions` in interface `weka.core.OptionHandler`


    - #### getUnion

      ```
      public int getUnion(java.util.TreeSet<java.lang.Integer> a,
                 java.util.TreeSet<java.lang.Integer> b)
      ```

      Calculate the size of the union of set A (feature attributes of instance
      A) and set B (feature attributes of instance B), that is, all the
      non-zero attributes the two instances have.

      Parameters:
      :   `a` -
      :   `b` -

      Returns:
      :   the size of the union of set A (feature attributes of instance A)
          and set B (feature attributes of instance B)


    - #### listOptions

      ```
      public java.util.Enumeration listOptions()
      ```

      **Specified by:**
      :   `listOptions` in interface `weka.core.OptionHandler`


    - #### postProcessDistances

      ```
      public void postProcessDistances(double[] distances)
      ```

      **Specified by:**
      :   `postProcessDistances` in interface `weka.core.DistanceFunction`


    - #### saveToFileAllDistances

      ```
      public java.lang.String saveToFileAllDistances()
      ```

      Generate and print distances for each instance vs each other instance.

      Returns:
      :   the path the distances were saved to


    - #### setAttributeIndices

      ```
      public void setAttributeIndices(java.lang.String value)
      ```

      **Specified by:**
      :   `setAttributeIndices` in interface `weka.core.DistanceFunction`


    - #### setInstances

      ```
      public void setInstances(weka.core.Instances instances)
      ```

      **Specified by:**
      :   `setInstances` in interface `weka.core.DistanceFunction`


    - #### setInvertSelection

      ```
      public void setInvertSelection(boolean value)
      ```

      **Specified by:**
      :   `setInvertSelection` in interface `weka.core.DistanceFunction`


    - #### setOptions

      ```
      public void setOptions(java.lang.String[] options)
                      throws java.lang.Exception
      ```

      **Specified by:**
      :   `setOptions` in interface `weka.core.OptionHandler`

      Throws:
      :   `java.lang.Exception`


    - #### update

      ```
      public void update(weka.core.Instance instance)
      ```

      **Specified by:**
      :   `update` in interface `weka.core.DistanceFunction`


    - #### main

      ```
      public static void main(java.lang.String[] args)
      ```


- Overview
- Package
- Class
- Use
- Tree
- Deprecated
- Index
- Help

- Prev Class
- Next Class

- Frames
- No Frames

- All Classes

- Summary:
- Nested |
- Field |
- Constr |
- Method

- Detail:
- Field |
- Constr |
- Method
